# SDN-Based Network Slicing Mechanism for a Scalable 4G/5G Core Network: A Kubernetes Approach †

**DOI:** 10.3390/s21113773

**Published:** 2021-05-29

**Authors:** Robert Botez, Jose Costa-Requena, Iustin-Alexandru Ivanciu, Vlad Strautiu, Virgil Dobrota

**Affiliations:** 1 Communications Department, Technical University of Cluj-Napoca, 400114 Cluj-Napoca, Romania; Iustin.Ivanciu@com.utcluj.ro (I.-A.I.); Vlad.STRAUTIU@student.utcluj.ro (V.S.); Virgil.Dobrota@com.utcluj.ro (V.D.); 2 Department of Communications and Networking, Aalto University, 02150 Espoo, Finland; Jose.Costa@aalto.fi

**Keywords:** 5G, cloud computing, EPC, IoT, Kubernetes, network slicing, NFV, SDN

## Abstract

Managing the large volumes of IoT and M2M traffic requires the evaluation of the scalability and reliability for all the components in the end-to-end system. This includes connectivity, mobile network functions, and application or services receiving and processing the data from end devices. Firstly, this paper discusses the design of a containerized IoT and M2M application and the mechanisms for delivering automated scalability and high availability when deploying it in: (1) the edge using balenaCloud; (2) the Amazon Web Services cloud with EC2 instances; and (3) the dedicated Amazon Web Services IoT service. The experiments showed that there are no significant differences between edge and cloud deployments regarding resource consumption. Secondly, the solutions for scaling the 4G/5G network functions and mobile backhaul that provide the connectivity between devices and IoT/M2M applications are analyzed. In this case, the scalability and high availability of the 4G/5G components are provided by Kubernetes. The experiments showed that our proposed scaling algorithm for network slicing managed with SDN guarantees the necessary radio and network resources for end-to-end high availability.

## 1. Introduction

The Internet of Things (IoT) has seen a major growth in recent years thanks to various applications emerging as a consequence of the evolution of smart sensors, artificial intelligence, robotics, or networking technologies. According to [1], Cisco predicts that half of the world’s connected devices will be represented by machine-to-machine (M2M) communications, reaching up to 14.7 billion connections or 1.8 M2M connections per inhabitant of the global population. Although in the previous report, M2M and IoT are considered two sides of the same coin, there is a difference between them. While the former is characterized by point-to-point communications with telemetry as the main application, the latter expand this capability by converging pools of M2M isolated systems over the Internet. The differences between these two technologies are seen in Table 1 [2].

Due to the fast growth of the industry, the amount of data generated by the entire IoT environment will be very high. All these data need to be stored, processed, and analyzed in a scalable infrastructure [3]. With its capacity to offer on-demand and scalable services, cloud computing is the perfect candidate for deploying IoT applications. Usually, applications are running on a virtualized infrastructure in the cloud. This approach has several benefits compared to using bare metal servers including reducing the capital and operating expenses (CAPEX and OPEX), increasing the security, efficiency, and scalability and solution recovery in case of disasters. Although bare metal servers are more suitable for mission-critical applications where latency is crucial, new optimization techniques make it possible to adopt virtualization technology in 5G industrial applications that require 1 ms round-trip time (RTT) [4].

Containerization technology has become a reliable solution for hosting applications in the cloud. A comparison between containerization and virtualization [5] has shown that containers have a higher scalability than virtual machines (VMs), making them a better alternative for IoT scenarios where scalability is important.

IoT applications come in all shapes and sizes and, as such, they must fulfill requirements in terms of long-range transmissions, costs, energy efficiency, latency, and bandwidth. The technologies that meet these requirements belong to the low-power wide-area network (LPWAN) domain. LPWAN technologies can be divided into two categories: cellular (LTE-M and NB-IoT) and non-cellular (Sigfox, LoRaWAN, and Symphony Link). The choice of using a specific LPWAN technology depends on the requirements that the IoT applications must meet; for example, Sigfox is the best option if the goal is to have a better coverage, while LoRa has the advantage of a flexible deployment. However, when it comes to applications where QoS, latency, scalability, and data rates are more important, NB-IoT is the way to go [6].

Nonetheless, the advent of the 5G technology should bring about effective interconnections between the vast numbers of IoT devices. The 5G technology standard [7] is tailored for three specific types of services:

- Enhanced mobile broadband (eMBB) will provide higher bandwidths and lower latency. It will be suitable for smart home applications, ultra-high-definition (UHD) television, or Cloud gaming services (for example, Google Stadia).
- Massive machine-type communications (mMTC) will improve the existing low-power wide-area networks (LPWANs). The purpose of this type of service is to pave the way for smart cities. The two LPWAN technologies used in 4G networks, NB-IoT and LTE-M, will still be used and will be important factors in meeting the requirements of the 5G standard for IoT.
- Ultra-reliable and low-latency communications (URLLC) is a special type of communications designed for mission-critical applications which require more bandwidth and lower latencies than mMTC. A specific case is illustrated in [8], where URLLC slices provide low latency for autonomous cars in an SDN-based core network (CN).

Taking into account the requirements for 5G networks and also the fact that this technology is designed for more than just mobile broadband [9], a new paradigm should be applied in backhaul and core networks. The requirements of latency and high availability for URLLC traffic could be resolved by enabling SDN in the backhaul network (BN). Moreover, the 5G core network has a service-based architecture (SBA), which means that the components could be implemented as software and deployed using network functions virtualization (NFV) as network functions (NFs). Using NFV management and orchestration (MANO) platforms for virtual network functions (VNFs) or network slices would lead to a 5G reliable core network. Moving the core components from physical dedicated servers to virtualized components running on a network function virtualization infrastructure (NFVI) brings many benefits not only in terms of capital and operational expenditure but also in terms of efficiency and scalability [10]. However, there are still some drawbacks, mainly because the VNFs are usually deployed in virtual machines. Thus, scaling, orchestrating, and managing these VMs for large-scale deployments becomes a difficult job due to the large overhead induced by virtualization. To overcome these limitations, migration to cloud-native network functions (CNFs) must be performed. CNFs [11] are mainly VNFs optimized to operate in a virtual cloud environment: they are based on microservices, hosted into containers and can be orchestrated with Kubernetes. Thus, reducing the overhead would be accomplished by containerization. Moreover, there are many advantages that CNFs can benefit from thanks to the intrinsic properties of Kubernetes such as autoscaling, high-availability, resilience, or built-in telemetry services.

This paper extends our previous work presented in [12]. As a first contribution, taken from [12], we compared the resource consumption of an IoT containerized telemetry application in different scenarios, with data processing performed either in the cloud (Amazon Web Services EC2 and Amazon Web Services IoT) or in the edge (balenaCloud). For a more accurate comparison, we used similar resources, the only difference being the hardware architecture for the edge deployment, which was ARM-based. The results were similar for resource consumption in terms of CPU and RAM memory. However, as we have seen in the previous paragraphs, 5G will be used for mMTC and other types of services, some of which are sensitive to latency. Thus, in 5G scenarios such as industrial IoT, not only the consumption of the computational resources must be taken into account, but also the efficiency of the network in terms of latency, throughput, and eventually packet-loss. As a second contribution, this paper proposes the usage of network slicing supported in 5G networks to separate radio, transport, and core network functions for different UEs with different traffic requirements. A novel solution of network slice scalability for managing NB-IoT traffic based on the least-load CNF, managed with SDN, is presented.

The paper is organized as follows. Section 2 presents an overview of the related work, and Section 3 describes the proposed architecture for two scenarios. The first scenario involves resource consumption for an IoT application deployed in balenaCloud, Amazon Web Services EC2 and Amazon Web Services IoT. The second scenario refers to the network slice scalability for managing IoT and MBB based on the least-load CNF managed with SDN and Kubernetes in a private cloud orchestrated by OpenStack. Section 4 presents the experimental results and Section 5 concludes the paper.

## 2. Related Work

Although technologies such as SDN and NFV have been present for some time, it is with the emergence of 5G that they will prove their true potential. First, they provide a financial advantage. In [13], a study was conducted to analyze the impact of using SDN, NFV and Cloud computing in 5G networks for the CAPEX, the OPEX and the total cost of ownership (TCO). It was observed that in comparison with the traditional architecture, the CAPEX would be reduced by 68%, the OPEX by 63%, and the TCO by 69%. Moreover, the development of NFV management and orchestration (MANO) platforms makes it easier to manage and orchestrate virtual network function (VNF) instances or network slices. This way, these technologies could help in implementing a reliable and scalable 5G core network. For example, there are some commercial solutions for 4G and 5G core networks on the market that rely on the previously mentioned technologies such as [14,15], or [16]. The cloud-native architecture of these deployments comes with some advantages, an important one being the ability to orchestrate and schedule the mobile core components on demand in order to deliver the required services with the proper quality of experience (QoE).

In [17], the requirements and challenges regarding the implementation of an EPC as a service in a cloud environment, along with the potential solutions to these challenges, are presented. The authors identified four types of mappings between the instances of EPC functions and the number of VMs for this approach, namely 1:1, 1:N, N:1, and N:2. These mappings are then compared, each having advantages and disadvantages when it comes to deploying the EPC as a service (EPCaaS). For example, although the 1:1 mapping has a simplistic architecture with each EPC component being hosted in a separate VM, other aspects such as scaling could be difficult to implement. Therefore, an appropriate mapping must be chosen according to the characteristics of services and applications using the EPC.

In [18], a solution to migrate the MME component into a cloud-native one, in order to implement dynamically auto-scaling, was proposed. The motivation for the paper was to minimize the overhead due to control signaling from IoT applications implemented over 5G networks. A comparison between the proposed MME scalable solution and the legacy one indicates that CPU consumption decreased by 26.64% and the throughput was higher by 7.19% for the scalable solution. Another solution for scaling the MME is the SCALE framework proposed in [19]. This framework also uses an 1:N mapping, which means that the MME needs to be redesigned by introducing two more components: an MME LoadBalancer (MLB), which forwards the traffic from UE to the specific MME, and the MME processing entity (MME), which stores information related to sessions and requests made by the UEs to the MME. Although the results are promising, redesigning the MME for real products can become problematic. Also, finding the correct MME within a pool for a specific request from a UE can increase the latency of the procedure. These limitations are overcome in a 5G core network by splitting the MME into three components: AMF, SMF, and UDM. In this approach, the AMF would be scaled, since it is the component which does the processing, while the UDM is hosting the UE contexts. This makes it possible for a UE to be served by any AMF connected to that UDM. An algorithm for scaling the AMF based on the load of the instances is presented in [20].

A solution to guarantee the resources for different traffic requirements and ensure a certain quality of experience is given by network slicing. One option for implementing network slices is based on the integration of SDN for managing the BN resources. In [21], a solution for providing URLLC using network slices in an SDN backhaul network is presented. The authors developed an algorithm that monitors the latency of the links in the backhaul topology and then computes the shortest path based on Dijkstra’s algorithm. Thus, the URLLC traffic will always be forwarded via the shortest path, ensuring the low latency requirement for this type of communication. In [22], another case for ensuring URLLC traffic with SDN is illustrated. In this paper, NB-IoT devices are allocated in the URLLC slice, where the low latency is maintained by policies implemented in the SDN controller. Moreover, with the increase of network congestion, the packet loss also increases for the non-URLLC slice. Aviable solution for creating the network slices in cloud environments is the integration of SDN with a network hypervisor. In [23], the Libera hypervisor was used to create a proof-of-concept model, called programmable network infrastructure as a service (p-NIaaS). This model provides an accessible way for tenants to program their allocated network infrastructure in a cloud infrastructure. By exposing the virtual NIs, the network slicing could be enabled with the use of SDN over the tenant virtualized network infrastructure.

Over the last years, alternatives to the classical OpenFlow-based switch Open vSwitch [24] were developed. One of them is the programmable virtual switch used in the Microsoft Azure public cloud, virtual filtering platform (VFP) [25]. VFP is based on match-action tables (MATs), which are implemented as layers to enable a multi-controller model, with each controller being able to apply its policy to the switch in a different layer. Another powerful solution used in public clouds is Orion [26], the SDN distributed platform developed by Google. One of the key aspects of Orion is the way it handles disconnect events coming from switches. It was observed that a disconnection event seen by the controller does not necessarily mean a dataplane failure. Thus, the strategy is to preserve the current state for larger and correlated failures, but to aggressively route if small and uncorrelated failures occur.

The measurement of the User Plane Function (UPF) performances in a real testbed environment is presented in [27]. This paper demonstrates the advantages of deploying the UPF as a standalone component for ensuring 5G requirements rather than in a monolithic 4G approach and uses Open Baton as an NFV MANO platform for orchestrating the core components as VNFs. The authors highlighted that having a large number of UPFs could lead to problems related to orchestration and management. Our proposed method can mitigate this limitation by deploying the core components as CNFs and orchestrate them with Kubernetes. Kubernetes, a mature container orchestration solution adopted in most cloud platforms, will ensure the efficient orchestration of a large number of CNFs, while the use of containers will significantly reduce the overhead previously introduced by virtual machines.

NFV platforms have started to develop rapidly with the advances in the field of 5G technology. Some of the existing MANO frameworks are used for deploying the 5G network functions, such as OSM [28], ONAP [29], OpenBaton [30], or Cloudify [31]. However, new MANO platforms have also been developed in order to meet 5G requirements. Many of these MANO platforms dedicated for 5G were developed within the 5G-PPP initiative [32]. One of them is SONATA [33], an NFV microservice-based platform. The insights for implementing SONATA are presented in [34]. The authors describe the main steps during the development phase of the platform and also how the DevOps approach improves the entire deployment process. Furthermore, at that time, they pointed out the gap in integrating the networking capabilities of Kubernetes in SONATA. Meanwhile, solutions for enabling Telco-grade network management in Kubernetes were developed, including Multus [35] and DANM [36]. Another NFV MANO platform developed within 5G-PPP is 5TONIC [37]. 5TONIC is more like an environment based on open technologies and designed specifically for the development of 5G technologies. The environment proposed by the authors has a local site, where there are two NFVIs managed by OpenStack and orchestrated by the OSM and to which external sites can also be connected. Thus, multiple external sites can be managed by a centralized MANO platform, which enables the automated deployment of network services (NSs) across them. In [38], a thorough comparison between MANO solutions dedicated for 5G is presented. The aim of the authors was to compare the SONATA platform with OSM and Cloudify. The results showed that SONATA outperformed OSM in terms of scaling-out time and Cloudify in terms of scaling-in time, but the NS instantiation time was longer than for the other two. All in all, the platforms responded well and had normal behavior during the experiments. The authors concluded that while OSM and Cloudify are very robust and can be used in a wide variety of cases, SONATA is a better tool for 5G scenarios where run-time service level agreement contracts and network slicing are involved.

## 3. Proposed System Architecture

This paper represents an extension of our previous work in [12] and aims to extend the previous experiments and also implement a testbed for evaluating the usage of network slicing across different mobile private operators. Our work is divided into two experiments covering the end-to-end system from services and applications to connectivity through mobile networks in order to deliver the data from end devices to the applications processing the data from those devices:

- The first experiment is related to a containerized telemetry application for IoT devices. We aim to analyze and compare the resource consumption of this application in three different deployment scenarios for both cloud and edge: (1) using a balenaCloud environment, (2) using AWS EC2 instances, and (3) using the AWS IoT cloud service. We chose these cloud environments because they can be used in a wide variety of deployment scenarios for IoT and M2M systems. balenaCloud is suitable for building and deploying containerized applications on remote devices in the edge. In AWS, we implemented the same application to perform a comparison in terms of resource consumption between cloud and edge. Third, Amazon Web Services IoT is one of the five major solutions with the largest market share alongside equivalent IoT platforms from Microsoft, Cisco, Google, and IBM [39]. Moreover, AWS IoT can be further integrated with AWS Wavelength for 5G deployments.
- The second experiment focuses on implementing an end-to-end testbed in order to deliver different types of traffic, with different traffic requirements through specific network slices when network congestion is detected. Moreover, the Mobile Private Cloud (MPC) network components are deployed as CNFs and orchestrated with Kubernetes. The motivation for using Kubernetes was to be able to provide high availability, scalability based on latency, and lifecycle management for the CNFs. This scenario aims to prove the feasibility and efficiency of our proposed algorithm for scaling the UPF and load-balancing the network traffic to the least-load CNF.

The division of the paper into two experiments was made in order to facilitate the better understanding of the topics. While the first experiment is related to the consumption of computational resources for an IoT application in different cloud environments, the second one extends the focus to the entire end-to-end system, from the sensors to the cloud, and provides a novel and optimal mechanism for managing IoT network slices over 4G/5G networks.

### 3.1. First Experiment: Monitoring the Resource Consumption of an IoT Telemetry Application in balenaCloud, Amazon Web Services, and Amazon Web Services IoT

In this first experiment, a DHT11 sensor that measures temperature and humidity was connected to a containerized application running on Docker. Three different scenarios were devised to better analyze the resource consumption of this application when using cloud- or edge-based IoT services. As such, the application was hosted: (1) locally, using balenaCloud, (2) in the AWS cloud using virtual machines, and (3) in the AWS cloud using the dedicated AWS IoT cloud service. Not only the hardware used but also the deployment methods vary between the three scenarios. For the first two cases, the DHT11 sensor was connected to an ESP32 development board. The gathered data were sent to an SQL database in AWS. For the third scenario, the sensor was connected to a Raspberry Pi 4, which forwarded the data to an InfluxDB database on the edge server. In all three scenarios, sensor data were displayed using Grafana. Finally, resource consumption monitoring was performed by means of CloudWatch and QuickSight for the AWS deployments, and Prometheus and Grafana for the edge deployment.

#### 3.1.1. balenaCloud

**Figure 1:**
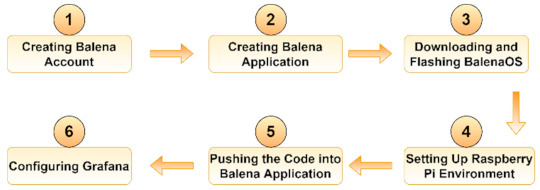
Creating and deploying a containerized IoT application using balena.

The main steps for creating and deploying the containerized IoT application using *balena* are presented in Figure 1.

**Figure 2:**
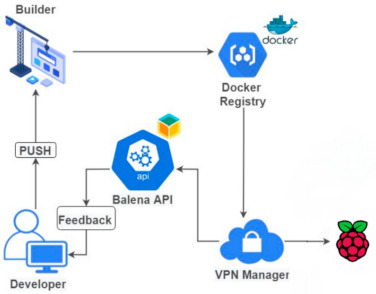
Proposed architecture in balenaCloud.

The first step was to create an account on the platform. As a device, we used a Raspberry Pi 4 with 4 GB RAM, which has the aarch64 hardware architecture supported by balenaCloud. Next, we added a specific balena entity, which contains both the fleet of IoT devices and the code running on it, as an application. Third, we had to download the balenaOS operating system and flash it to an SD card using an open-source cross-platform tool, called balenaEtcher. Connecting the device to the platform is initially done by means of a provisioning key, which is deleted from the device after the first boot. From then on, another unique key, received via VPN, is used. Managing the fleet can be done either via the dashboard or the dedicated API with balena CLI and SDK. The code running on the fleet is sent to the build server validation. Here, Docker images are built according to the specific hardware of the devices that has to be specified in Dockerfiles. These images are then forwarded to a private container registry and downloaded to the receiving device, as illustrated in Figure 2.

The data gathered from the DHT11 sensor were sent to an InfluxDB database running on a local server, rather than the Raspberry Pi. We chose this approach not only because we wanted to ensure the scalability of the solution (having a server allows the collection of data in a centralized manner), but also because we did not want the added workload to interfere with our resource consumption measurements. The metrics regarding resource utilization were gathered by a Prometheus agent and displayed in Grafana.

#### 3.1.2. Amazon Web Services

**Figure 3:**
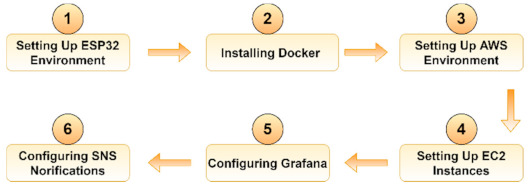
Creating and deploying a containerized IoT application using AWS.

The main steps for creating and deploying the containerized IoT application in AWS are presented in Figure 3.

**Figure 4:**
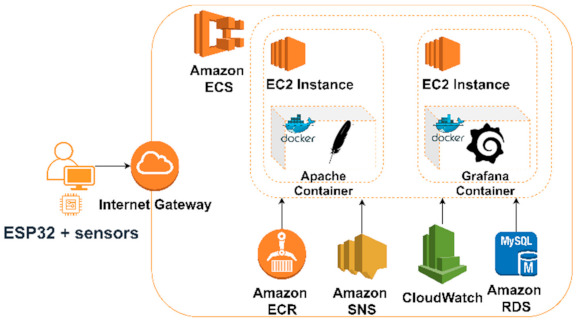
Proposed architecture in Amazon Web Services.

In this approach, the first step was to program the ESP board using the open-source Arduino IDE with the ESP32 add-on. Moreover, it is important to note that in the first scenario a dedicated Docker daemon, balenaEngine, was available by default. In this second scenario however, the Docker daemon needed to be installed. We used two containers running on two different EC2 instances: one container was responsible for collecting the data and sending it to the database using an Apache server, while the second container ran Grafana. The proposed architecture in AWS is illustrated in Figure 4.

As for the first scenario, Grafana was configured to display both the data gathered from the sensors and the metrics related to resource monitoring gathered by CloudWatch.

#### 3.1.3. Amazon Web Services IoT

**Figure 5:**
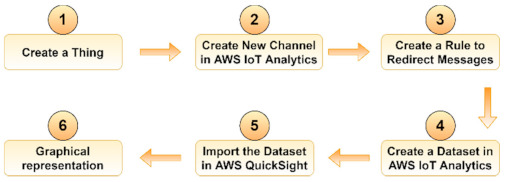
Creating and deploying a containerized IoT application using AWS IoT.

For a better comparison with the previous two implementations, we decided to present the main steps for deploying the application using AWS IoT (Figure 5). However, the architecture used is the default one provided by AWS, described in detail in [40], and was therefore not included herein for brevity.

Data gathered by the sensors were collected in AWS IoT Analytics. The raw, unprocessed messages are archived into a channel which stores all data from a certain MQTT topic. The MQTT protocol was created especially for low latency and small-sized packets characteristic of IoT devices. As such, compared to the previous implementation using HTTP, MQTT can ensure high delivery guarantees and has higher throughput and lower energy consumption and bandwidth usage. Moreover, it is suitable for intermittent connections. Messages from a channel can be redirected by the IoT Core to AWS IoT Analytics by means of user-defined rules. The dataset is imported into AWS QuickSight for graphical representation. The main advantage of using AWS IoT is that the serverless infrastructure is managed entirely by AWS and, as such, this solution requires the least time and effort to be deployed.

### 3.2. Second Experiment: Network Slice Scalability Managed with SDN and Kubernetes in a Private Cloud Orchestrated by OpenStack

For the second scenario, we developed a mechanism which not only improves the scalability of our solution, but also optimizes the end-to-end communication in terms of latency and throughput in a 4G/5G network. When deploying the mobile network functions we opted for a private cloud orchestrated by OpenStack, due to the following reasons: (1) balena uses its own operating system, balenaOS, which is an optimized operating system for IoT devices, the core network that we used required some Debian-specific dependencies to function properly; (2) even if, in theory, a private cloud is more expensive than a public one, the equipment we already own are sufficient for running the experiments, and thus no additional cost was involved; (3) OpenStack was our choice for orchestrating the private cloud because it is an open-source solution and a reference as a virtualized infrastructure manager (VIM) [17] for NFV MANO. It is already the first choice as a VIM in platforms such as ONAP or OSM.

**Figure 6:**
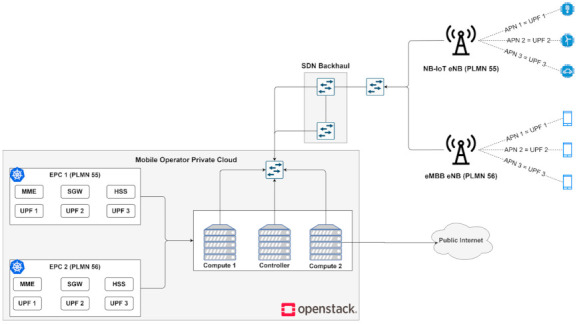
The proposed system architecture for the second scenario.

The proposed architecture is illustrated in Figure 6. The Radio Access Network (RAN) is composed of two eNBs, which serves two different types of traffic: IoT and mobile broadband. The backhaul network is represented by two OpenFlow Open vSwitch switches which connect the RAN to the CN. All the traffic is received by the first switch and forwarded to the CN, if there is no congestion. If congestion occurs, the traffic originating from the eNB designated with the IoT traffic will be forwarded to the second switch, and from there to the CN. This way, two network slices are enabled by the use of SDN: one for IoT traffic and the other one for mobile broadband. Finally, in the cloud, we deployed two CNs, each one representing a different mobile operator. We deployed EPC components as CNFs with the use of Kubernetes. Using a 3-node K8s cluster, not only the scalability and lifecycle management but also the high availability for the CNFs is ensured.

#### 3.2.1. Testbed Description

**Figure 7:**
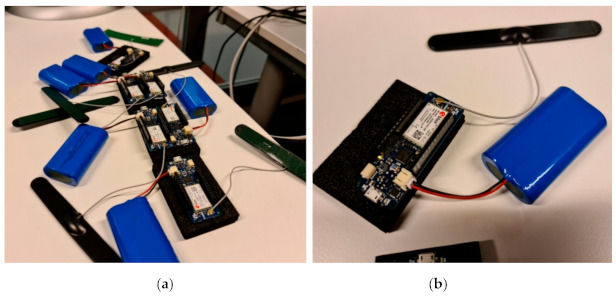
Experimental testbed for NB-IoT devices. (**a**) With six u-blox NB-IoT chipset. (**b**) With one u-blox NB-IoT chipset.

The testbed consists of NB-IoT devices and mobile phones as UE. We used Arduino boards with the u-blox NB-IoT chipset (Figure 7), a Bittium Tough Mobile, and a Motorola Moto C Plus. It is worth mentioning that the operation mode for NB-IoT is in-band, which means that the sensors are using the same radio access network and share the same spectrum resources as the mobile terminals.

These devices were connected to two different Nokia FZM commercial eNBs: one running on 1.8 GHz for NB-IoT and the other one running on 2.6 GHz is serving the mobile phones. The configuration of the eNBs is presented in Table 2.

Next, the eNBs are connected to an L2 switch, which connects the RAN with the backhaul network. The backhaul consists of two OvSs installed on an Ubuntu 18 server which isolate the IoT traffic from the MBB traffic when congestion occurs. The switches from the backhaul are directly connected with the Mobile Operator Private Cloud.

**Figure 8:**
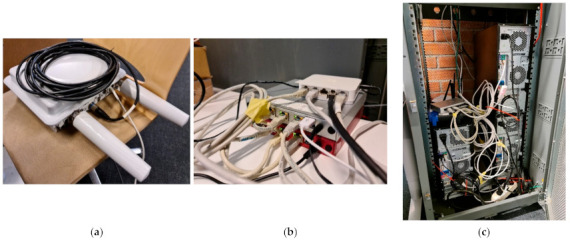
E2E experimental testbed. (**a**) Nokia FZM eNB. (**b**) Open vSwitches. (**c**) OpenStack private cloud.

The private cloud was deployed on three HP Z240 Tower Workstations. In the logical architecture in OpenStack one of the nodes is the controller and the other two are compute nodes. On top of Kubernetes, we deployed a three-node Kubernetes cluster where we deployed the CNs. Then, we deployed the packet core on the Kubernetes cluster. Also, we deployed the HSS separately using an Ubuntu 18 cloud instance. The AMF and the SMF were implemented as deployment resources with a single pod, using Multus for assigning static IP addresses. Even if we do not use the replication feature for these two deployments because of the static IP configuration, using a deployment object instead of a standalone pod ensures high availability by creating the same pod component in cluster in case of a node or a pod failure. The entire experimental testbed, which is conceptually displayed in Figure 6, is illustrated in Figure 8: one of the Nokia FZM eNBs that we used is illustrated in (a), the OvS switches used in the backhaul are illustrated in (b) and the physical infrastructure of the private cloud orchestrated by OpenStack is shown in (c).

#### 3.2.2. Migration to Cloud-Native Network Functions

**Figure 9:**
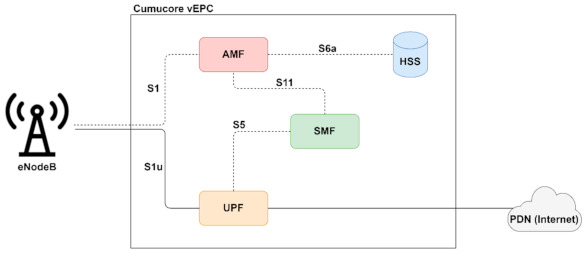
Cumucore vEPC architecture.

In this paper, we used the commercial Cumucore 4G/5G packet core for the CN. The architecture of this packet core is illustrated in Figure 9. Although we used a 4G core network, because of the Cumucore packet core architecture we will refer to the components in the control plane as AMF and SMF and to the user plane component as UPF. Moreover, the proposed solution in this paper can also be used in a 5G core.

The Cumucore vEPC [15] is an optimized solution for private mobile networks, research testbeds, corporate mobile networks, or small/medium operators. Having a microservice-based architecture, it fits perfectly for cloud-native deployments. The official documentation presents the installation process of this solution to a single VM with an Ubuntu 18 server. However, this approach does not fit our purposes. Hosting the entire core in a single VM makes it impossible to scale different components of the core on-demand. Moreover, a single instance of the EPC represents a single point-of-failure of the entire system. Even so, taking advantage of the microservice-based architecture of this solution, we separated each component into a different virtual network function. Still, in order to have control over the entire core, we need a central orchestrator to implement functions such as high availability, scalability, lifecycle management, monitoring or troubleshooting for the components. One solution would be to adopt an NFV MANO platform such as ONAP or OSM for orchestrating the core components such as VNFs. However, using a different virtual machine for each component would lead to a higher waste of resources and a higher overhead and would also increase the system delay when creating new instances. A different approach would be to implement the components as CNFs, which seems to be more appropriate since the core already has a microservice architecture. Moving the core components to containers means lower overhead, lower startup time, and higher portability. Moreover, orchestrating the containers with Kubernetes will ensure all the features mentioned in the previous paragraph. Since the orchestration of CNFs in most MANO platforms already requires a Kubernetes cluster over which these should run, we decided to use Kubernetes as a MANO platform and take advantage of its innate properties.

The containerization of the packet core is not a trivial task. The first step was to identify all the dependencies that each component needs to build the Docker images. The Docker images are used to create Docker containers and are created based on a Dockerfile for each core component. Each Dockerfile represents a script that is used to provide the Docker daemon with information about how the image should be built including the base image, the dependencies, environmental variables, executing startup commands, and so on. Note that even though the containers are running on the host OS and the default user inside containers is the root, it is not the same root as on the host. This is a good security feature, because the root user inside the container is mapped on the host as a non-root user, thus not being able to take control over the host capabilities in case of attacks such as privilege escalation. Moreover, this information is important because, by default, Docker starts the containers with a limited set of capabilities. However, the UPF component must use the GPRS Tunneling Protocol (GTP) kernel module on the host to establish the GTP tunnel with the eNB for carrying the user data traffic. Considering these aspects, the UPF containers need to run in privileged mode. A security context needs to be created for UPF pods to add kernel capabilities to them. This way, the UPF pods are able to use the GTP kernel module on the host. The security context can be set so that the containers inside the pods run in the privileged mode, in which case all the host kernel capabilities will be available for the container. To manipulate only the network stack on the host and implicitly the GTP module, the NET_ADMIN and NET_RAW capabilities must be set inside the security context of the pods. Although this aspect raises some security issues, studying them is out of the scope of this paper.

Orchestrating the containerized core network with Kubernetes is also challenging. First, the EPC architecture requires that all the components are stateful, and this implies a serious scalability problem: the IP addresses of the pods are not known by the developer, being assigned to the pod just after it was scheduled. Considering this, an L4 or an L7 load balancer is usually employed as a single endpoint for a set of pods. But having multiple replicas of MME could lead, for example, to one UE being served by another MME for different requests. The same problem also arises for the other components; for example, if a GTP tunnel is established between the eNB and the UPF, but the UE will be forwarded by the Kubernetes load balancer to another UPF pod, then the PDN connectivity will be lost. As pointed out in the Related Work section, the EPC components had to be modified by introducing some databases for storing their states to achieve on-demand scalability.

**Figure 10:**
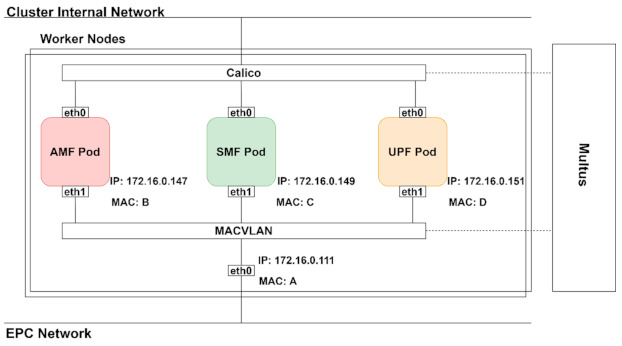
Kubernetes networking.

In this paper, we want to achieve scalability without modifying the components, since we use a commercial packet core. Although scaling the AMF still implies changes in the component by saving the state of every AMF in a database, we can achieve scalability for the UPF. For this, we need to preserve the stateful behavior of the components. Also, the connection between components by well-defined interfaces (e.g., S5, S11) must be preserved. As a result, attaching different interfaces to pods must be done, besides the default network providing cluster internal addresses assigned by Kubernetes. Enabling multiple container network interfaces (CNI) in Kubernetes can be achieved with Multus. Thus, the default CNI (in our case, Calico) provided by Kubernetes for the pods will be used to maintain the connection with the Kubernetes server, while the other interfaces assigned by Multus will be used to interconnect the packet core components (Figure 10).

For the additional container network interface (CNI) enabled by Multus, we used the Macvlan driver. This way, the node network interface is split into multiple IP addresses and MAC addresses to the pods. Assigning other interfaces to EPC pods is important so that they are visible to the eNB.

#### 3.2.3. Scaling Algorithm for UPF

Next, we proposed a method to scale the UPF using native Kubernetes mechanisms, without modifying the component. The UPF component was also implemented as a Kubernetes deployment resource but, in order to enable the replication feature, the IP addresses of the second interfaces of the pods will be assigned automatically from a pool of IP addresses. This is due to the fact that it is not possible to provide static IP allocation to a Replica Set resource.

To scale the UPF pods, we used the Horizontal Pod Autoscaler (HPA) resource. The HPA uses Metrics API to provide the measurements based on which the scaling will be done. By default, the HPA uses for scaling just metrics related to CPU and Memory utilization. However, we were interested instead in scaling the UPF based on the incoming bit rate on the UPF network interface. This could decrease the latency due to the queueing delay when the incoming bit rate is higher than the capacity of the interface, by forwarding the traffic from the new incoming users to a new UPF. To send more metrics to the HPA, another API must be created in Kubernetes. For this, we deployed the v1beta1.external.metrics.k8s.io API to send the number of bits per second received on the UPF pod interface to Kubernetes. We used Kube Metrics Adapter [41], an open-source project, for implementing the custom and external metric APIs. Then, Prometheus was deployed in the existing cluster, so that the HPA could collect the measurements from the v1beta1.external.metrics.k8s.io API and do the scaling based on Prometheus metrics.

To scale the UPF pods, the HPA will detect when a specified threshold set for the bits per second measurement is exceeded. The rule used by the Kubernetes HPA for the scaling decision is

(1) $$dR=\lceil cR\times \frac{cM}{dM}\rceil$$

where *dR* represents the desired number of pods, *cR* is the current number of pods, *cM* is the measured metric, and *dM* is the threshold specified for the metric. The ceil function also ensures the existence of at least one UPF pod in the system.

Let us consider (1) for the scaling up operation. If we consider *N* to be the number of current UPF pods and the current measurement as the mean measured load value for the *N* components, then (1) becomes (2) and, after simplification, we obtain (3):

(2) $$dR=\lceil N\times \frac{\frac{1}{N}\times \sum_{i=1}^{N}cM_{i}}{dM}\rceil$$

(3) $$dR=\lceil \frac{\sum_{i=1}^{N}cM_{i}}{dM}\rceil$$

and for every real *x* and *y*, the following property for ceil function holds:

(4) ⌈x+y⌉≤⌈x⌉+⌈y⌉

Thus, (3) becomes

(5) $$dR=\lceil \frac{\sum_{i=1}^{N}cM_{i}}{dM}\rceil \leq \lceil \frac{cM_{1}}{dM}\rceil +\lceil \frac{cM_{2}}{dM}\rceil +\ldots +\lceil \frac{cM_{N}}{dM}\rceil$$

Now, we assume that the threshold set for the desired metric value is at least half of the total capacity of the network interface, each UPF has open connections with UEs, and the new incoming traffic is only sent to one UPF replica at a time. Thus, the ceil function for each component is defined by (6):

(6) $$\lceil \frac{cM_{k}}{dM}\rceil =\left\{\begin{array}{c} 0,cM_{k}=0\\ 1,cM_{k}\in \left(0,dM\right]\\ 2,cM_{k}\in \left(dM,TotalCapacity\right) \end{array}\right.$$

Consequently, based on the traffic load for each component, the following inequality is valid for the desired number of UPF components:

(7) $$dR\leq \left\{\begin{array}{l} N-1,cM_{k}=0\\ N,cM_{k}\in \left(0,dM\right]\\ N+1,cM_{k}\in \left(dM,TotalCapacity\right) \end{array}\right.\;\mathrm{where}\left\{\begin{array}{l} k\in \left[1,N\right]\\ dM\in \left(\frac{TotalCapacity}{2},TotalCapacity\right) \end{array}\right.$$

Given the fact that the new incoming traffic is sent to the most recently created UPF, we considered the inequality (7) as an equality in the following cases:

(8) $$dR=\left\{\begin{array}{l} N-1,where\;cM_{k}=0\;for\;k\neq N\\ N,\forall cM_{k}\neq 0\;and\;at\;least\;one\;cM_{k}<dM\\ N+1,\forall cM_{k}\geq dM \end{array}\right.$$

**Figure 11:**
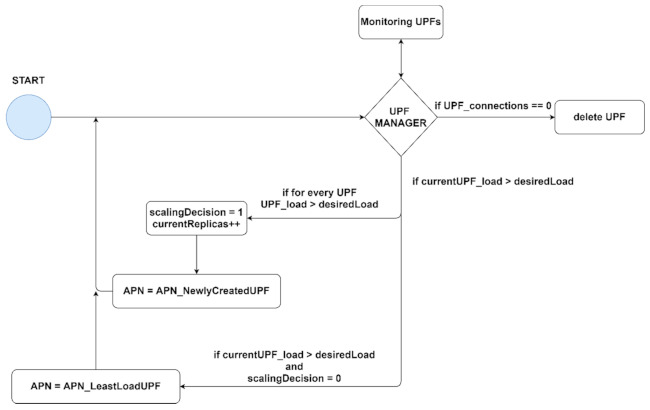
The proposed scaling algorithm for UPF.

The previous equation illustrates how the scaling decision must be made in order to have the optimal number of UPFs in the system. Thus, the scaling must be done only when the measured metric is above the threshold for all the replicas. Based on (8), we proposed the following scaling algorithm, illustrated in Figure 11.

We implemented an entity, named UPF Manager (UPFM), as an orchestrator for UPF components. UPFM continuously queries metrics provided by Prometheus to measure the network load. The idea was to extend the existing HPA scaling capabilities and customize them for our specific needs. A threshold for the network load of the UPF was set and when this threshold is exceeded, another UPF to which new connected UEs will be forwarded is created. If more than one UPF is created, using only the HPA capabilities could, in some cases, lead to high load. This aspect will be discussed in more detail in the next section.

For every UE and UPF, their information is stored in the HSS with a specific Access Point Name (APN). This way, the UE will connect to the UPF with the same APN. To ensure uninterrupted communication and avoid a reattachment procedure of the UE, when the new UPF is created, this is stored with the APN of the previous UPF which is overloaded. Consequently, the UEs that are using the previous UPF will still be using it for existing sessions. A list with the available UPFs is also stored in the UPFM for the following reason: when the current UPF is overloaded but the load of a previous UPF is below the threshold, the UPFM should forward the new incoming traffic to it, thus preserving the current number of UPFs. The number of pods should only be scaled up when all of the existing pods from the list are overloaded. Also, if a pod from the list has no more connections, it must be deleted since no other traffic will be forwarded to it.

Since we do not know the precise IP address assigned by Multus to the newly created UPF, a script is triggered after the pod’s creation with the use of container lifecycle hooks. This will send its IP address and the host name to UPFM together with a flag. A flag is also sent when a UPF is deleted to know which UPF must be removed from the list. For this, we exposed an API on the UPFM to listen for pod requests triggered by the creation or deletion of a container.

#### 3.2.4. Network Slicing for Different Types of Network Traffic

**Figure 12:**
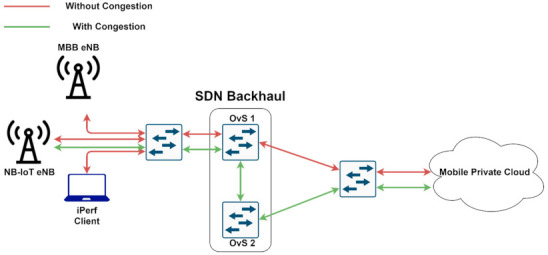
Network Slicing enabled by SDN for IoT and MBB traffic.

Another aspect that must be considered is separating customer and IoT network traffic in the backhaul network. The idea of separation is to prevent one type of traffic from interfering with the other, in the case of heavy traffic situations. To accomplish this, we create network slices for different types of traffic based on congestion measurements, with the use of SDN. In our scenario (Figure 12), the traffic from both eNBs is multiplexed through one L2 switch and is then sent to the backhaul network. We used two OpenFlow switches for the backhaul network, implemented with the use of Open vSwitch: if there is no congestion between backhaul and MPC, then the first OvS will be used for all the network traffic. However, if network congestion is detected, then the IoT traffic is sent to the cloud via the second OvS. In this way, we are able to create different network slices for separating customer and IoT traffic in case of network congestion. For measuring the latency, we used an active measurement technique between OvS 1 and the MPC: we sent ICMP packets once a second and measured the Round-Trip Time (RTT). Then, we set a threshold for the RTT and when the threshold is exceeded, a rule based on the source MAC address for the incoming packets is set on the OvS 1 in order to forward the IoT traffic to the OvS 2 and from there to the cloud.

There are multiple solutions for monitoring network metrics in an SDN topology, a popular one being [42], which enables accurate measurements for delay, packet-loss or available transfer rate for links and switches. However, in our scenario, only the OvS 1 needs to be managed to assign different paths for every type of network traffic. Moreover, since there is only one path by default between eNBs and MPC, we assumed that the forward and backward paths were the same in terms of congestion when it occurred. Thus, the end-to-end delay (i.e., latency) was approximated as half of the RTT. The ICMP packets sent for the delay measurement were captured in OvS 1 with tcpdump, which added timestamps to them. When the sequence number of an ECHO REQUEST matched the one of an ECHO REPLY, the delay was computed every second by subtracting the timestamps from these packets. Then we verified whether two consecutive computed delays were above the first set threshold of a hysteresis. If only the first RTT was above the threshold (and not the next one) no action was required. If both RTTs exceeded the threshold, the OvS 1 started to forward the IoT traffic through another path until two consecutive RTTs fell below the second threshold. This hysteresis is useful to avoid route oscillations. The forwarding is done when one of the above two actions occurred by deleting and adding a new path for the packets incoming and destined for the MAC address of the eNB IoT. All the operations described above were performed by a Python script which interacted with Linux processes for running tcpdump by opening a pipe with the popen() method. Next, the ICMP packet processing was done in real time, and the computed RTTs were saved in a Python List. When the script detected congestion, it interacted with the ovs-ofctl program to add the flows into the OvS 1 flow table.

## 4. Experimental Results

In this section, we present the results for the two scenarios discussed in the previous paragraphs.

### 4.1. Experimental Results for the First Scenario

**Figure 13:**
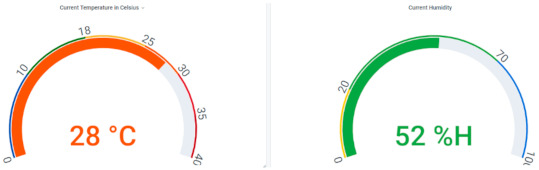
Dashboard corresponding to temperature and humidity.

Two Grafana dashboards were created for the balenaCloud and AWS implementations: one was used to monitor the values gathered by the IoT sensors, while the second was used to monitor the resource consumption of the containerized application. Temperature and humidity are displayed on the first dashboard, as illustrated in Figure 13.

**Figure 14:**
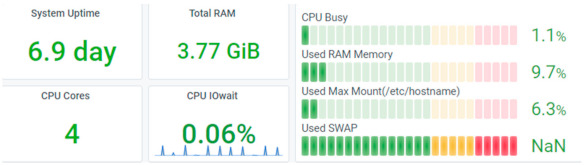
Resource consumption of Raspberry Pi in balenaCloud.

**Figure 15:**
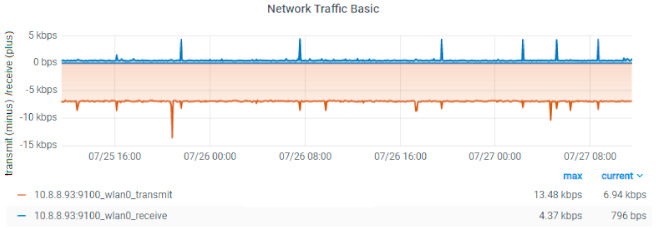
Network traffic for the Raspberry Pi in the balena implementation.

Depending on the implementation, resource consumption monitoring was performed in the cloud or on the Raspberry Pi. Using balenaCloud, all the computation is done on the edge device (Raspberry Pi), while for the two AWS-based implementations the ESP32 is just a gateway, and the computation is done on the EC2 instances. Data related to resource consumption in the first scenario were gathered via Prometheus and displayed in Grafana, as illustrated in Figure 14 and Figure 15.

The application running on the Raspberry Pi uses 1.1% of the four CPU cores and 9.7% of the 4 GB RAM memory. By comparison, the AWS cluster composed of EC2 instances uses 4.45% of 1 vCPU and 2.4% of 3 GB cluster RAM memory (1 GB per instance). The higher memory consumption for the Raspberry Pi is due to the fact that the AWS instances run only strictly necessary services and are highly optimized in this sense. Note that the Raspberry Pi transmits more data than it receives. This is not unexpected since we configured the device as a gateway for collecting data from the sensors and forwarding it to balenaCloud. The only traffic received is the signaling sent from the cloud which also includes commands for the remote control of the device.

**Figure 16:**
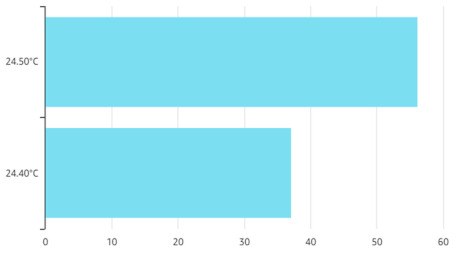
Number of counts for temperature in QuickSight.

**Figure 17:**
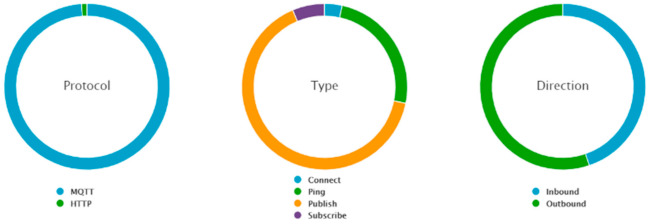
AWS IoT Core message statistics.

In AWS IoT, data are represented graphically using QuickSight. A count of records by temperature is illustrated in Figure 16. As explained in the previous section, AWS IoT Core uses messages to communicate with the IoT devices. AWS IoT is based on serverless functions; therefore, a comparison in terms of resource consumption cannot be performed with the first two scenarios. Instead, the cost is determined by the number of requests. Several statistics are readily available, as illustrated in Figure 17.

Most of the messages were MQTT and outbound, corresponding to data publishing. However, there were also some HTTP messages used for connecting. Other types of messages were used to periodically check the connectivity with ping or to subscribe to a certain channel.

### 4.2. Experimental Results for the Second Scenario

**Figure 18:**
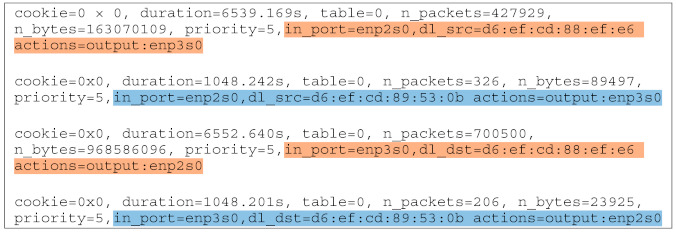
Flow table for OvS 1 before network congestion.

**Figure 19:**
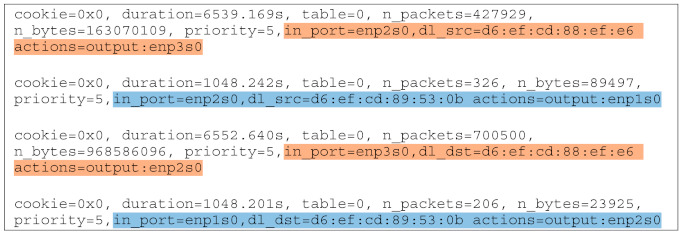
Flow table for OvS 1 after network congestion.

As can be seen in Figure 12, OvS 1 is responsible for isolating traffic in case of congestion. Thus, an application was developed in order to program this switch for forwarding traffic in case of congestion based on eNB MAC addresses. Figure 18 and Figure 19 illustrate the flows before and after the congestion occurs. Note that for the MBB eNB (D6:EF:CD:88:EF:E6) the flow path highlighted in orange remains the same, while for the IoT eNB (D6:EF:CD:89:53:0B) both the input and output ports are modified to assign a different slice and consequently a different flow path, highlighted in blue.

**Figure 20:**
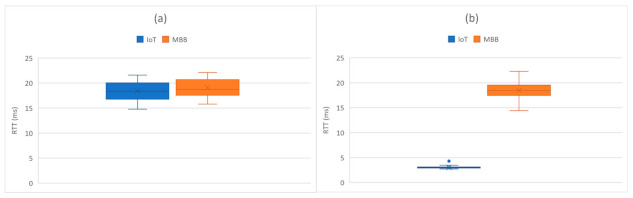
Comparison in RTT variation between IoT and MBB traffic: (**a**) when using the same link; (**b**) after the network congestion detection feature is enabled.

**Figure 21:**
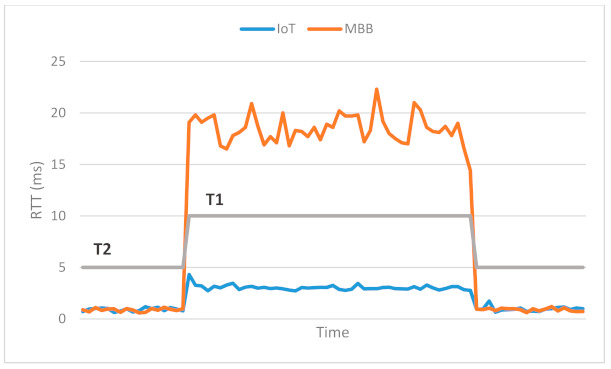
Comparison in RTT between IoT and MBB slices.

Thus, the traffic for IoT devices served by the eNB with MAC address D6:EF:CD:89:53:0B was isolated. The congestion was detected using an active measurement technique, inspecting the RTT on the link between OvS 1 and the cloud. The first congestion threshold was set to 10 ms and the second one to 5 ms. The difference in RTT between the IoT (blue) and the MBB (orange) slices is illustrated in Figure 20 and Figure 21. We first sent all the traffic through the same link to highlight the motivation for traffic isolation. The variation of RTT for both IoT and MBB, when using the same link, is illustrated in Figure 20a, while Figure 20b presents the variation of RTT when the OvS is using the congestion detection feature. It is important to mention that the values are based on measurements between OvS 1 and the UPFs and do not reflect the end-to-end latency. This is because we wanted to evaluate the effect of isolating the traffic in different slices in the backhaul network with SDN. Since the slices are created in the backhaul, adding the latency from the radio network would not lead to a correct assessment. Figure 21 also illustrates the two thresholds, T1 set to 10 ms and T2 set to 5 ms, which define the hysteresis for the congestion detection.

It can be observed that there is a significant difference between the RTT values for the IoT and MBB traffic in case of congestion when network slices were created. While the mean value of RTT for the IoT slice is 3.0456 ms, the same value for the MBB slice is 18.4673 ms. Moreover, as it can be seen in Figure 12, the network traffic from both eNBs is multiplexed into a L2 switch, which could be a bottleneck. However, when different ports were used to connect both eNBs to OvS, the RTT for the IoT traffic was below 1 ms.

**Figure 22:**
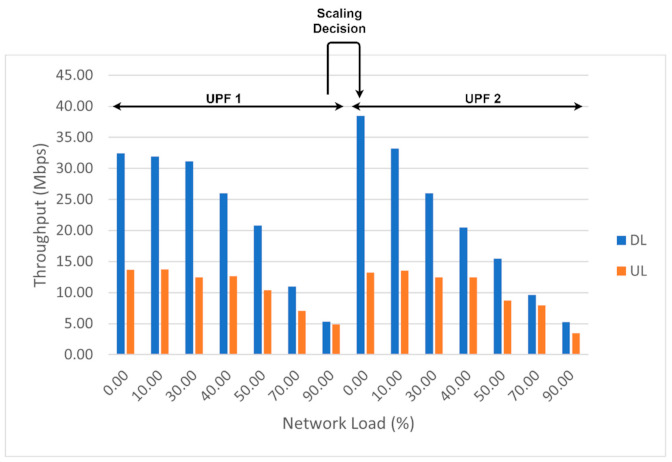
Throughput variation for UE with UPF network load affected by the scaling decision.

Next, we evaluated our proposed scaling algorithm for UPF. For this, we started congesting the existing UPF with UDP traffic using iperf and measured the end-to-end throughput directly from the UEs, using the Speedtest application for Android [43]. We evaluated the throughput of UEs by congesting the UPF at different network load thresholds (0%, 10%, 30%, 40%, 50%, 70%, and 90%). We observed in some cases that the drop of downlink throughput is linear with network load increase, and in other cases the decreasing slope becomes steep after the 40% threshold. These variations could be introduced by radio interference or by the Speedtest application we used for estimating the end-to-end throughput. We ran multiple tests between the Motorola smartphone and the Speedtest application using a UPF with a network load within 10%, and the range for variations within 5 Mbps. For 10 consecutive measurements, the results varied between 30.86 and 34.72 Mbps with a mean value of 31.94 Mbps. The range for the Bittium smartphone was different, i.e., 37.67 and 41.33 Mbps. The variations do not seem unusual, especially for an end-to-end system that involves radio connectivity. Nonetheless, up to the threshold of 40% we observed a throughput greater than 20 Mbps on DL and 12 Mbps on UL, and a reduction to 10 Mbps on DL and under 10 Mbps on UL when the threshold exceeded 70%. Figure 22 illustrates the scaling decision of our algorithm, using a threshold of 90%. Once the network load exceeded this threshold, another UPF was created and used by the newly attached UE.

**Figure 23:**
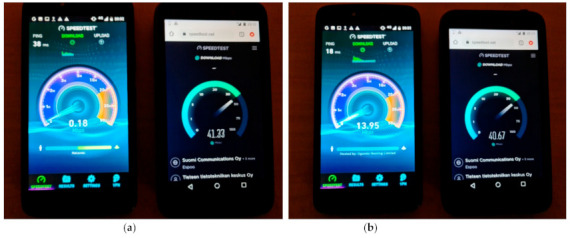
The throughput difference for the UEs connected to different UPFs. (**a**) The load of the previous UPF is almost 100% (left image). (**b**) The load of the previous UPF is approximate 70% (right image).

The difference in throughput when the Motorola smart phone (left) is connected to the previous overloaded UPF and the Bittium smart phone (right) is connected to the newly created UPF is illustrated in Figure 23a,b. In Figure 23a, the previous UPF has almost a load of 100%, while in Figure 23b the network load is 70%.

**Figure 24:**
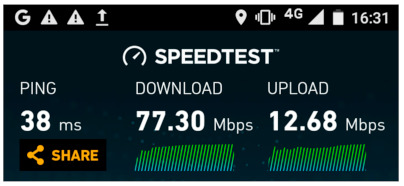
The maximum throughput for Motorola Moto C Plus connected to the MBB eNB.

In these experiments, the mobile phones were connected to the NB-IoT eNB to allow for a visual comparison (Figure 23) in terms of UE throughput achieved by using our proposed algorithm. The reason why we did not use the other eNB was because the Bittium smart phone cannot use the 38 LTE band used by that eNB. However, the behavior and the results for our scaling algorithm would be the same; only the numerical values at different thresholds for throughput would be different. The difference in throughput when using the other eNB can be seen in Figure 24. Here, the Motorola smart phone was used to show the maximum achievable throughput (77.3 Mbps for DL and 12.68 Mbps for UL) for our deployment in the 38 LTE band.

**Figure 25:**
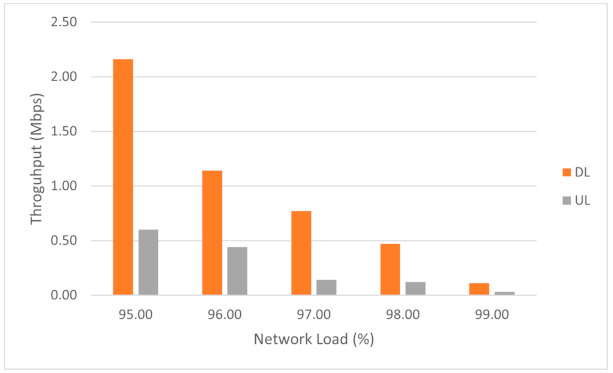
The UE throughput variation in case of high network load on UPF.

**Figure 26:**
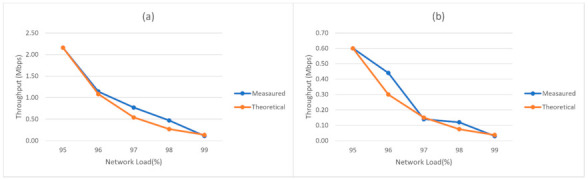
Comparison of UE throughput between the measured value and the value predicted by (9) in case of network load higher than 95%. (**a**) Downlink. (**b**) Uplink.

We also measured the UE throughput for very high UPF loads to evaluate its behavior. The results are depicted in Figure 25:

We observed that the reduction of the UE throughput after the 95% threshold can be approximated by an exponential decay law, halved with increasing the load with 1%, as described in (9):

(9) $$\theta \left(95+x\right)=\theta_{0}\times 2^{-x}$$

where $\theta \left(95+x\right)$ represents the UE throughput as a function of network load for network loads over 95%, $\theta_{0}$ represents the UE throughput at 95% UPF load, and *x* represents 1% of the UPF network load. An interesting fact is that we can make an analogy with the half-life period in radioactive decay, if we consider 1% of UPF load as the half-life period [44]. The difference between the measured values and theoretical values approximated by (9) for the UE throughput is illustrated in Figure 26a for DL and Figure 26b for UL. Although there is a slight difference in the case of a few values, it is important to note that the measurements performed at these loads are unstable since it is very difficult to maintain a constant value of the load.

**Figure 27:**
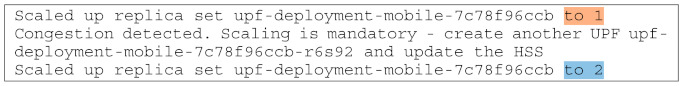
Scaling decision in case of high load on UPF.

**Figure 28:**
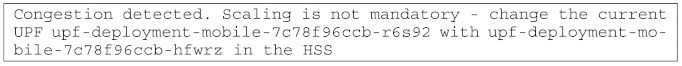
Load-balancing decision in case of high load on UPF.

To provide a better understanding of our mechanism, we illustrate the log messages for two different scenarios (see Figure 27 and Figure 28): (1) the UPF load is higher than the threshold and because there is just one pod (orange), another one needs to be created (blue); and (2) even though the current UPF load is higher than the threshold, a new session can be accommodated in another active UPF. Hence, there is no need to scale up the pods.

The behavior of our mechanism was compared with the usage of the default Kubernetes HPA. While for the first scenario presented in Figure 27 the behavior was the same, a big difference was observed for the second scenario. After the second UPF was created, we congested it to compare the default HPA mechanism with the one we developed. For this, we set up the threshold to 60% of the UPF capacity. The main disadvantage of the default HPA is using the mean value for all the pods, and this was illustrated in the next three experiments.

For the first experiment, we considered the first UPF congested with 20% network load above the threshold. In this case, both mechanisms scaled to three replicas when the HPA detected the mean value of the network load being above the threshold. For the second experiment, we decreased the congestion for the first UPF to 30% below the HPA threshold and then we started to congest the second UPF. In this case, our algorithm started to forward the traffic to the first UPF (as in Figure 28), while the default HPA waited until the mean value of the network load was exceeded. For the default mechanism to scale up, we needed to congest the second UPF with 30% above the set threshold. Also, the default HPA created another pod in this case, while our approach starts to load-balance the traffic to the old one if its capacity can serve other UEs. Finally, in the third experiment, we decreased the congestion for the first UPF to 50% of its capacity. The default HPA becomes unusable since our theoretical model requires that the desired metric after which the HPA has to scale must have a value between half and full of the UPF capacity. In this case even if we congest the second UPF up to its full capacity, the mean value will remain at 55% and the HPA will never scale up. The effect was that the same congested UPF was used for the incoming UEs, while for our approach the same behavior was observed as in Figure 28.

## 5. Discussions and Conclusions

This paper extends our previous work in [12] related to the resource consumption of containerized applications for IoT in various deployments. We propose a novel mechanism for scaling network slices in order to forward NB-IoT and MBB traffic using the least-load CNF managed with SDN and Kubernetes. Since in our previous paper we focused on CPU and RAM memory utilization, in this work the network load and delay were analyzed in order to optimize the connectivity for both the IoT and MBB traffic. We consider this a logical follow-up given the enormous amount of information that is estimated to be provided by IoT devices in 5G networks and beyond.

First, an application was implemented for dynamically assigning network slices for IoT and MBB traffic in case of congestion. It was observed that by using different slices, we significantly reduced the RTT for the IoT traffic in case of link congestion, from 18.4673 ms to almost 3 ms. A disadvantage of our implementation is that we used a switch to multiplex the traffic from the two base stations in a single link, which could affect the end-to-end delay in case this link gets congested. To tackle this drawback, traffic from eNBs should not be multiplexed, but sent on different physical links instead.

Next, we considered implementing a 4G/5G core network using an NFV approach. Since the majority of NFV MANO platforms are VNF-based, we chose Kubernetes for a containerized approach. Thus, a commercial 4G/5G packet core was deployed on a Kubernetes cluster in order to orchestrate its components and ensure features such as high-availability, scalability, monitoring, or lifecycle management. With this CNF approach, we wanted to scale the UPF capacity by load-balancing the incoming traffic through multiple UPFs using a least-load policy. For this, we proposed a scaling algorithm and implemented a new component, namely UPFM, which overcomes the Kubernetes HPA limitations when it comes to external metrics. One limitation that the default HPA has with external metrics is that scaling can be done by evaluating either the current or the average value. Using the current value leads to evaluating only the load of the first created pod. The average value could be used, but there are some situations that can lead to overloading the UPF without making the scaling decision. The experimental results for such scenarios were presented in the previous section and proved the advantages that our algorithm brings.

Our proposed scaling algorithm performs optimal scaling, the scaling decision being made only when all the available UPFs are almost overloaded. When no scaling is needed, the UPF with the least load is selected from the available ones and the incoming traffic is forwarded to it. Considering the previous case, our algorithm evaluates whether the current UPF exceeds the threshold, and if so, it will check the load of the other UPFs to evaluate if they can serve more UEs. Next, the incoming traffic will either be balanced to the least-load one, or forwarded to a newly created UPF. Forwarding is done by updating the HSS with the IP address of the chosen UPF. A limitation of our algorithm is that we cannot scale down the components given the current behavior of the HPA. To reduce the number of UPFs we have to check whether an unused available UPF has active sessions or not and as such can be deleted. The HPA cannot scale down as the UPF that serves active sessions, because it does not know how to eliminate a certain pod. A solution would be to create a custom Kubernetes HPA that makes the scaling down decision based on the number of GTP tunnels which can help us determine if there are more users attached. We have this idea in mind for further implementation. The results showed that our algorithm is efficient, making the scaling decision based on a set threshold. We demonstrated that after the load of the current UPF is exceeded, another load-less UPF is created in order to serve further connections with a maximum throughput. Moreover, we demonstrated that if the scaling is not necessary, the load balancing is done to the least-load available UPF.

Future work will consider the implementation of a custom HPA Kubernetes, which will add the scaling-down capability to our algorithm. Another consideration is to design a system for traffic anomaly detection based on a supervised learning method, in order to identify whether a CNF is loaded and needs scaling up. Based on its performances, this could be a valuable extension of our current algorithm and might even replace it entirely.

## Figures and Tables

**Table 1:** Comparison of M2M and IoT.

M2M	IoT
Point-to-point communications	Connectivity via IP networks
Hardware-based technology	Suitable for both hardware and software
Not dependent on the Internet	Relies on Internet connectivity
Device-based communications	Interface devices with gateways or data systems
Limited scalability	Scalability is a key requirement

**Table 2:** Configuration mode for eNBs.

Scope	LTE Band	Duplex Scheme	UL Carrier Frequency	DL Carrier Frequency	Channel Bandwidth	Carrier Power	MAC Address
IoT	3	FDD	1720 MHz	1815 MHz	10 MHz	24 dBm	D6:EF:CD:89:53:0B
MBB	38	TDD	2610 MHz	2610 MHz	20 MHz	24 dBm	D6:EF:CD:88:EF:E4

## Data Availability

The data that support the findings of this study are available from the corresponding author, R.B., upon reasonable request.

## References

[1] Cisco Annual Internet Report (2018–2023) White Paper. https://www.cisco.com/c/en/us/solutions/collateral/executive-perspectives/annual-internet-report/white-paper-c11-741490.html.

[2] Presad R., Rohokale V., Prasad R., Jackson O. (2020). Internet of Things (IoT) and Machine to Machine (M2M) Communication. Cyber Security: The Lifeline of Information and Communication Technology.

[3] Marjani M., Nasaruddin F., Gani A., Karim A., Hashem I.A.T., Siddiqa A., Yaqoob I. (2017). Big IoT Data Analytics: Architecture, Opportunities, and Open Research Challenges. IEEE Access.

[4] Xiang Z., Gabriel F., Urbano E., Nguyen G.T., Reisslein M., Fitzek F.H.P. (2019). Reducing Latency in Virtual Machines: Enabling Tactile Internet for Human-Machine Co-Working. IEEE J. Sel. Areas Commun..

[5] Zhang Q., Liu L., Pu C., Dou Q., Wu L., Zhou W. A Comparative Study of Containers and Virtual Machines in Big Data Environment. Proceedings of the IEEE 11th International Conference on Cloud Comput. (CLOUD).

[6] Mekki K., Bajic E., Chaxel F., Meyer F. (2019). A comparative study of LPWAN technologies for large-scale IoT deployment. ICT Express.

[7] The Path to 5G: As Much Evolution as Revolution. https://www.3gpp.org/news-events/1774-5g_wiseharbour.

[8] Chekired D.A., Togou M.A., Khoukhi L., Ksentini A. (2019). 5G-Slicing-Enabled Scalable SDN Core Network: Toward an Ultra-Low Latency of Autonomous Driving Service. IEEE J. Sel. Areas Commun..

[9] Yu H., Lee H., Jeon H. (2017). What is 5G? Emerging 5G Mobile Services and Network Requirements. Sustainability.

[10] Basta A., Blenk A., Hoffmann K., Morper H.J., Hoffmann M., Kellerer W. (2017). Towards a Cost Optimal Design for a 5G Mobile Core Network Based on SDN and NFV. IEEE Trans. Netw. Serv. Manag..

[11] Cloud-Native Network Functions. https://www.cisco.com/c/en/us/solutions/service-provider/industry/cable/cloud-native-network-functions.html#~introduction.

[12] Botez R., Strautiu V., Ivanciu I.-A., Dobrota V. Containerized Application for IoT Devices: Comparison between balenaCloud and Amazon Web Services Approaches. Proceedings of the 2020 International Symposium on Electronics and Telecommunications (ISETC).

[13] Bouras C., Ntarzanos P., Papazois A. Cost modeling for SDN/NFV based mobile 5G networks. Proceedings of the 8th International Congress on Ultra-Modern Telecommunications and Control Systems and Workshops (ICUMT).

[14] Enabling Next Generation Mobile Networks. https://cumucore.com/#products.

[15] Evolve Your Core Network for 5G. https://www.ericsson.com/en/core-network/5g-core.

[16] 5G Core (5GC). https://www.nokia.com/networks/portfolio/5g-core/#5g-core-solution.

[17] Taleb T., Corici M., Parada C., Jamakovic A., Ruffino S., Karagiannis G., Magedanz T. (2015). EASE: EPC as a service to ease mobile core network deployment over cloud. IEEE Netw..

[18] Amogh P.C., Veeramachaneni G., Rangisetti A.K., Tamma B.R., Franklin A.A. A cloud native solution for dynamic auto scaling of MME in LTE. Proceedings of the IEEE 28th Annual International Symposium on Personal Indoor, and Mobile Radio Communications (PIMRC).

[19] Banerjee A., Mahindra R., Sundaresan K., Kasera S., Van der Merwe K., Rangarajan S. Scaling the LTE control-plane for future mobile access. Proceedings of the 11th ACM Conference on Emerging Networking Experiments and Technologies.

[20] Alawe I., Hadjadj-Aoul Y., Ksentini A., Bertin P., Darche D. On the scalability of 5G core network: The AMF case. Proceedings of the 15th IEEE Annual Consumer Communications & Networking Conference (CCNC).

[21] Hefele A., Costa-Requena J. SDN managed Network Slicing in Mobile Backhaul. Proceedings of the International Conference on Electronics Information, and Communication (ICEIC).

[22] Adem A., Costa-Requena J., Kantola R. SDN Network Slicing for URLLC NB-IOT. Proceedings of the International Conference on Electronics Information, and Communication (ICEIC).

[23] Yang G., Yu B.-y., Jin H., Yoo C. (2020). Libera for Programmable Network Virtualization. IEEE Commun. Mag..

[24] Open vSwitch. https://docs.openvswitch.org/en/latest.

[25] Firestone D. VFP: A Virtual Switch Platform for Host SDN in the Public Cloud. Proceedings of the 14th USENIX Symposium on Networked Systems Design and Implementation.

[26] Ferguson A.D., Gribble S., Hong C.Y., Killian C., Mohsin W., Muehe H., Ong J., Poutievski L., Singh A., Vicisano L. Orion: Google’s Software-Defined Networking Control Plane. Proceedings of the 18th USENIX Symposium on Networked Systems Design and Implementation Virtual Conference.

[27] Costa-Requena J., Poutanen A., Vural S., Kamel G., Clark C., Roy S.K. SDN-Based UPF for Mobile Backhaul Network Slicing. Proceedings of the 2018 European Conference on Networks and Communications (EuCNC).

[28] Open Source MANO. https://osm.etsi.org.

[29] Open Network Automation Platform. https://www.onap.org.

[30] OPEN BATON. https://openbaton.github.io.

[31] Cloudify. https://cloudify.co/technologies/.

[32] The 5G Infrastructure Public Private Partnership. https://5g-ppp.eu.

[33] SONATA. https://5g-ppp.eu/sonata/.

[34] Soenen T., Van Rossem S., Tavernier W., Vicens F., Valocchi D., Trakadas P., Karkazis P., Xilouris G., Eardley P., Kolometsos S. Insights from SONATA: Implementing and Integrating a Microservice-based NFV Service Platform with a DevOps Methodology. Proceedings of the NOMS 2018-2018 IEEE/IFIP Network Operations and Management Symposium.

[35] Multus-CNI. https://github.com/k8snetworkplumbingwg/multus-cni.

[36] DANM. https://github.com/nokia/danm.

[37] Nogales B., Vidal I., Lopez D.R., Rodriguez J., Garcia-Reinoso J., Azcorra A. (2019). Design and Deployment of an Open Management and Orchestration Platform for Multi-Site NFV Experimentation. IEEE Commun. Mag..

[38] Trakadas P., Karkazis P., Leligou H.C., Zahariadis T., Vicens F., Zurita A., Alemany P., Soenen T., Parada C., Bonnet J. (2020). Comparison of Management and Orchestration Solutions for the 5G Era. J. Sens. Actuator Netw..

[39] Making Sense of IoT Platforms: AWS vs. Azure vs. Google vs. IBM vs. Cisco. https://www.altexsoft.com/blog/iot-platforms/.

[40] AWS Documentation. https://docs.aws.amazon.com.

[41] Kube Metrics Adapter. https://github.com/zalando-incubator/kube-metrics-adapter.

[42] Van Adrichem N.L.M., Doerr C., Kuipers F.A. OpenNetMon: Network monitoring in OpenFlow Software-Defined Networks. Proceedings of the 2014 IEEE Network Operations and Management Symposium (NOMS).

[43] Speedtest. https://www.speedtest.net.

[44] Rutherford E. (1900). A radio-active substance emitted from thorium compounds. Lond. Edinb. Dublin Philos. Mag. J. Sci..

